# Exploring the structure and dynamics of proteins in soil organic matter

**DOI:** 10.1002/prot.26070

**Published:** 2021-03-25

**Authors:** Mathias Gotsmy, Yerko Escalona, Chris Oostenbrink, Drazen Petrov

**Affiliations:** ^1^ Department of Material Sciences and Process Engineering, Institute of Molecular Modeling and Simulation University of Natural Resources and Life Sciences Vienna Vienna Austria

**Keywords:** complex environments, molecular dynamics simulation, protein‐solvent interactions, soil organic matter

## Abstract

Alongside inorganic materials, water, and air, soil organic matter (SOM) is one of the major components of soil and has tremendous influence on the environment given its vital role in the carbon cycle. Many soil dwelling organisms like plants, fungi and bacteria excrete proteins, whose interaction with SOM is poorly understood on an atomistic level. In this study, molecular dynamics simulations were used to investigate selected proteins in soil models of different complexity from simple co‐solvent molecules to Leonardite humic acids (LHA). We analyzed the proteins in terms of their structural stability, the nature and strength of the interactions with their surroundings, as well as their aggregation behavior. Upon insertion of proteins in complex SOM models, their structural stability decreased, although no unfolding or disruption of secondary structure was observed. The interactions of proteins and SOM were primarily governed by electrostatic forces, often in form of hydrogen bonds. However, also weaker van der Waals forces made a significant contribution to the total interaction energies. Moreover, we showed that even though the molecular structure and size of SOM molecules varied, the functional groups of SOM ordered around the protein in a similar pattern. Finally, the number of aggregates formed by proteins and SOM molecules was shown to be primarily proportional to the size of the latter. Strikingly, for varying protein net charges no changes in the formation of aggregates with the strongly negatively charged LHA were observed.

## 1 INTRODUCTION

Soil organic matter (SOM) is defined as the product of organic molecule degradation processes in soil. Its major components are decomposing plant parts, microbial remains, mineral‐bound organic matter, charcoal from forest fires, as well as dissolved organic matter.^1^ Even though SOM constitutes only a small fraction of soil, it has great influence on many of its properties.^2^ Amino acids, peptides, and proteins compose a large fraction of SOM and contain most of the nitrogen in soil. Their origin is 2‐fold: (a) certain specific proteins are purposefully excreted into the soil by some organisms in order to fulfill specific functions while (b) the majority of proteins in SOM are released after cell death.^3^ Their preservation in soil organic matter can be attributed to copolymerization,^4^ adsorption,^5^ and encapsulation.^6^

There is scientific effort to understand the interactions of proteins in SOM at a structural level. Insecticidal and infectious proteins, specifically Cry toxin and prions, have been of particular interest due to their potential toxicity and pathogenicity, which may cause disease in humans and animals.^6^, ^7^ Additionally, the gained understanding could accelerate the improvement process of enzymes for bioremediation applications.^4^, ^8^ Moreover, the presence of SOM has a positive impact on the health of plants. This is due to various interactions of SOM molecules and plant derived biomolecules in the rhizosphere.^9^ The investigation of SOM‐protein interactions in the rhizoshpere can help to explain the nature of these interactions and how they affect the whole plant organism.

However, due to the high complexity of SOM, it is difficult for researchers to compare and reproduce their results. A solution to this problem is the definition of humic substance standard samples that are used as SOM models by researches worldwide. To date, several studies have utilized humic substances standard samples and model proteins to gain insight into protein‐SOM interactions.^5^, ^6^, ^7^, ^10^, ^11^

In recent years, atomistic models have been developed to shed light on the molecular interactions of soil components. Molecular dynamics (MD) simulations allow us to study the dynamics of these models as well as their molecular interactions with proteins.^12^, ^13^, ^14^, ^15^, ^16^ Computational analysis of such complex systems is opposed by challenges arising from the size of the proteins and the description of their interactions with SOM through force fields. Most proteins active in soil are large, require co‐factors, and/or form complexes with other proteins. However, small reference proteins can be employed in MD simulations to reduce the computational load. Additionally, to create models that can represent the complexity of SOM a tool called Vienna Soil Organic Matter Modeler (VSOMM; https://somm.boku.ac.at/)^17^, ^18^ has been devised. VSOMM uses small organic fragments as building blocks to create molecular SOM models. These building blocks contain different amounts of carbon, oxygen, nitrogen and sulfur and were designed to reflect a high diversity in the number and chemical properties of functional groups. Additionally, the total number of building blocks per system and the number of building blocks per molecule can be adjusted. Currently, the second generation (VSOMM2) presents several improvements, particularly within the implementation of a broader set of building blocks, which increases the chemical and geometric diversity of the models.^18^ Using VSOMM, several recent computational studies were able to reproduce the results of wet‐lab experiments.^19^, ^20^, ^21^, ^22^, ^23^ Moreover, VSOMM uses the GROMOS 54A7 force field which initially was parameterized for proteins which allows us to accurately describe proteins and SOM molecules in a combined MD simulation.^24^

In this work, in order to study the interactions between protein and humic substances, two proteins (villin and spitz) were selected as reference proteins from a subset of well‐known and previously characterized proteins in our group.^25^, ^26^, ^27^ These proteins were simulated within SOM models of increasing complexity. Initially, we tested the interaction of proteins with simple organic co‐solvents to assess the effects of different organic compounds or moieties in SOM, including carboxyl groups or aromatic rings. Subsequently, we used SOM models of Leonardite humic acids (LHA) since such models have been shown to yield realistic observations and to reproduce experimental solvation free energies of small compounds.^19^, ^20^ This study expands our knowledge about protein‐SOM interactions and will facilitate future exploration of SOM molecules with other biomolecules, especially in the context of enzyme engineering for bioremediation, understanding the effect of preservation of toxic proteins in soil as well as understanding the plant rhizosphere.

## 2 METHODOLOGY

### 2.1 Reference proteins

The villin headpiece domain of chicken (*Gallus gallus*; PDB 1VII)^28^ and the EGF domain of spitz of *drosophila melanogaster* (PDB 3CA7)^29^ were used as reference proteins. The selection considered following criteria: (a) small size to ensure short simulation times (villin 36, spitz 50 amino acids); (b) different protein net charges (villin +2, spitz −2) to study possible differences of interactions between the protein and the strongly negatively charged humic acids; (c) different secondary structures (villin only *α*‐helices, spitz *α*‐helices and *β*‐sheets) to investigate different protein structures.

### 2.2 Experimental design

Each reference protein was simulated in water, in four different simple co‐solvent systems (which represented various properties of soil organic matter), and additionally in more complex and realistic SOM models. The simple co‐solvent systems were: (a) calcium chloride (CaCl_2_), (b) calcium acetate (CaAcet_2_), (c) calcium benzoate (CaBenz_2_), and (d) SOM‐like, which is combination of the previous co‐solvents to mimic the Leonardite humic acid functional group composition. The systems were neutralized with Ca^2+^ ions and solvated with explicit SPC water. All systems were simulated in aqueous environments with H_2_O mass fractions of 0.74 and more, which is well above what has been previously reported as minimum for a water activity of 1.^19^

For the more complex SOM models, preequilibrated systems of Leonardite humic acid created by the Vienna Soil Organic Matter Modeler 2 (VSOMM2) were used. All models contained the same total number of building blocks (200) comprising the humic acid molecules, but the number of building blocks (BBs) per molecule (2, 5, 10, and 20 BBs per mol) was altered to observe differences related to molecular size. Table 1 lists the relative frequencies of functional groups per carbon atom for all simulated systems. A comparison with experimental data of LHA samples provided by the International Humic Substance Society (IHSS)^30^ is given in the last row.

**TABLE 1:** Carbon fractions of functional groups of the organic compounds in the simulated systems

Name	BB/mol	Carbon fractions						Avg. MW
		Carbonyl	Carboxyl	Aryl	Acetal	Heteroaliphatic	Aliphatic	
H_2_O	–	–	–	–	–	–	–	–
CaCl_2_	–	–	–	–	–	–	–	–
CaAcet_2_	1	–	0.50	–	–	–	0.50	59
CaBenz_2_	1	–	0.14	0.86	–	–	–	121
SOM‐like	1	–	0.17	0.67	–	–	0.16	69
SOM‐2	2	0.08	0.14	0.59	0.04	0.01	0.14	267
SOM‐5	5	0.08	0.14	0.59	0.04	0.01	0.14	671
SOM‐10	10	0.08	0.14	0.59	0.04	0.01	0.14	1339
SOM‐20	20	0.08	0.14	0.58	0.04	0.01	0.14	2602
IHSS LHA Sample^30^		0.08	0.15	0.58	0.04	0.01	0.14	–

*Note*: As a comparison, the last line shows data given by IHSS for the LHA carbon fractions.^30^

Abbreviations: Acet, acetate; Avg. MW, average molecular weight of organic co‐solvent molecules in g/mol; BB/mol, number of building blocks per molecule; Benz, benzoate; LHA, Leonardite humic acid.

### 2.3 Insertion of protein into LHA systems

**FIGURE 1:**
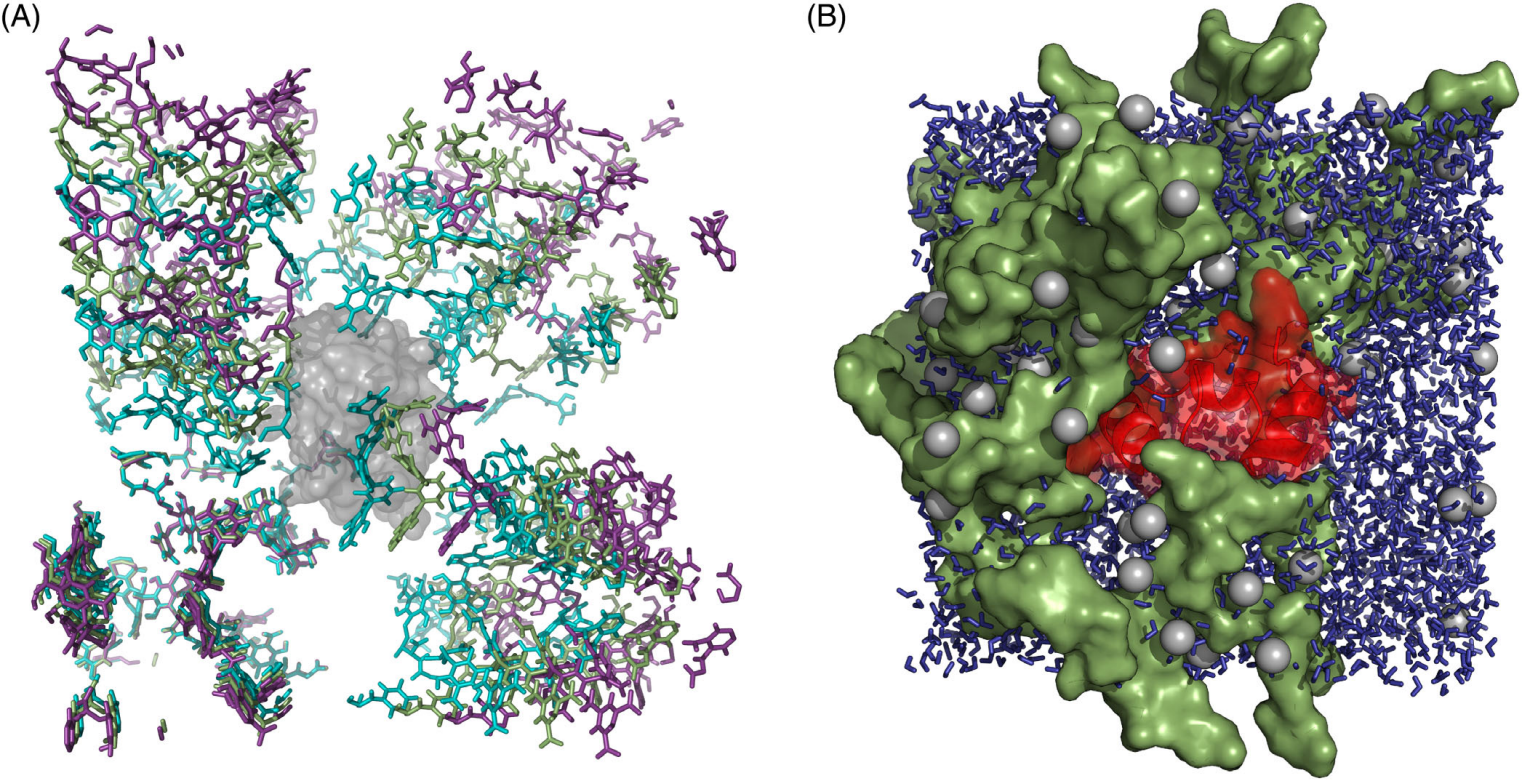
A, Three frames of the VSOMM2 systems protein insertion protocol. The protein (villin) is represented in gray. The most inflated system state is shown in purple. Half way and complete deflation to the original box size are shown in smudge green and teal, respectively. B, Rendering of villin in an equilibrated SOM‐10 system. The protein is shown in red, SOM molecules in smudge green, water molecules in blue and Ca^2+^ ions in gray. The molecular structures were rendered with PyMol.^42^ [Color figure can be viewed at wileyonlinelibrary.com]

Due to the heterogeneity of the SOM systems and its compacted structure, it was necessary to insert the reference proteins into the LHA matrix. The methodology used was based on the InflateGro method proposed by Kandt et al.^31^ However, due to the differences between inserting a membrane peptide into a lipid bilayer and proteins into soil organic matter, several adjustments had to be made. To insert the reference proteins into the prepared systems, the systems were initially inflated by +1 nm in three dimensions and the protein was added. The systems subsequently were deflated in 10 steps (−0.1 nm per step), each followed by an energy minimization simulation, back to its original box size. The energy minimization was done by the steepest descent algorithm while positionally restraining the protein with a force constant of 10^5^ kJ/(mol nm^2^). A visual representation of the method and its application for protein insertion is shown in Figure 1A.

### 2.4 Molecular dynamics

All simulated boxes were subsequently processed with tools of GROMACS version 2019.1.^32^ The molecular topology files were created with the 54A7 GROMOS force field.^24^ The systems were solvated using default van der Waals radii^33^ and subsequently neutralized by the replacement of water molecules with Ca^2+^ ions. An energy‐minimization step was performed using the steepest descent algorithm to a force lower than 10^3^ kJ/(mol nm). An atomistic cutoff‐scheme was used for all molecular dynamics simulations with a cut‐off for electrostatic and van der Waals forces at 1.4 nm. An additional reaction‐field contribution to the energies and forces with a dielectric permittivity of 61 was applied. Equilibration was performed in two distinct steps of 100 ps simulation each, starting with an NVT simulation. The leap‐frog algorithm was used for integration with a step size of 2 fs. The protein was positionally restrained with a force constant of 10^3^ kJ/(mol nm^2^). All bonds were constrained with the LINCS algorithm.^34^ The temperature was restrained at 300 K using a weak coupling thermostat^35^ with three different temperature groups (protein, SOM, water + monoatomic ions) and a coupling time of 0.1 ps. The velocities were initially assigned according to a Maxwell‐Boltzmann distribution. The second step of equilibration is a NPT molecular dynamics simulation. Simulation parameters stayed the same, except for the addition of an isotropic weak coupling barostat.^35^ The coupling parameter was set to 0.5 ps and the isothermal compressibility of water to 4.5 × 10^−5^ bar^−1^. The molecular dynamics simulation was performed for 100 ns with the same settings except that the positional restraints of the protein were removed. After 20 ns an equilibrium by convergence of the potential energy was observed and hence the last 80 ns of each run were used for the analysis. All simulations were performed in triplicates with different random number seeds for the generation of initial velocities. A rendering of the SOM‐10 simulation is depicted in Figure 1B.

### 2.5 Trajectory analysis

Different analyses were performed on the simulated trajectories to understand the structure and dynamics of the systems. Unless stated otherwise, they were carried out using the GROMACS analysis tools.^32^ In order to measure the proteins' structural stability, the root‐mean‐square fluctuations (RMSF) of the position of the C*α* atoms and the positional root‐mean‐square deviation (RMSD) of the backbone atoms with respect to the PDB structure as reference were calculated. To examine the conformational similarity of the protein trajectories in different SOM model systems, the pairwise harmonic ensemble similarities (*D*_*HES*_) were determined according to Lindorff‐Larsen and Ferkinghoff‐Borg^36^ using the ENCORE Python package.^37^ To do so, trajectories of both proteins were pre‐processed by writing out snapshots every 40 ps and concatenating replicates. Additionally, the average fraction of the secondary structure of protein was calculated using the GROMACS implementation of the DSSP algorithm.^38^ To understand the forces governing interactions between proteins and their surroundings, the nonbonded interaction energies were calculated. The interaction energies were grouped according to different solvent components (water, cations, anions, and benzene). To gain insight into the proximity of different functional groups or ions of co‐solvent molecules to the protein, a minimum distance function (MDF) was calculated. The MDF is defined as the distance between the closest two atoms from two previously specified groups of atoms for every frame of an MD trajectory. For every condition the MDF was calculated between every co‐solvent functional group (carboxyl, aryl) or ion (Ca^2+^, Cl^−^) and the protein. MDFs of the same functional group or ion type in the same condition were subsequently concatenated and transformed into a histogram (200 bins). The histogram frequency was then normalized by the number of concatenated functional groups or ions in the system. To complement the MDF observations, the number of hydrogen bonds were calculated and averaged over the simulated trajectories. The default values for hydrogen bond definition were used. In this analysis, we did not distinguish whether the protein was donating or accepting a hydrogen bond from its surroundings. To quantify and describe the phase separation that occurred in some of our systems, the preferential solvation between different species was calculated via Kirkwood‐Buff integrals for the simple co‐solvent systems. The preferential solvation (*δ*) values were calculated for solvent‐only systems of the conditions CaCl_2_, CaAcet_2_, CaBenz_2_, and SOM‐like (100 ns, 1 replicate) according to Ben‐Naim (1989).^39^ The Kirkwood‐Buff integrals were averaged between 1 and 1.6 nm distance to the molecule. For the SOM models, a cluster analysis was done based on the hydrogen bond connectivity between humic substances molecules. Two molecules were defined to be part of the same cluster if at least one hydrogen bond was connecting them. Only SOM and protein molecules were considered for this analysis. Statistical pairwise comparisons of protein properties in SOM conditions to water were performed with Student's *t* test (with n = 3 for both samples) and corrected for multiple tests using the Bonferroni method. Following significance levels were used throughout this publication: * for *P* ≤ .05, ** for *P* ≤ .01, and *** for *P* ≤ .001.

## 3 RESULTS

### 3.1 Protein stability

**FIGURE 2:**
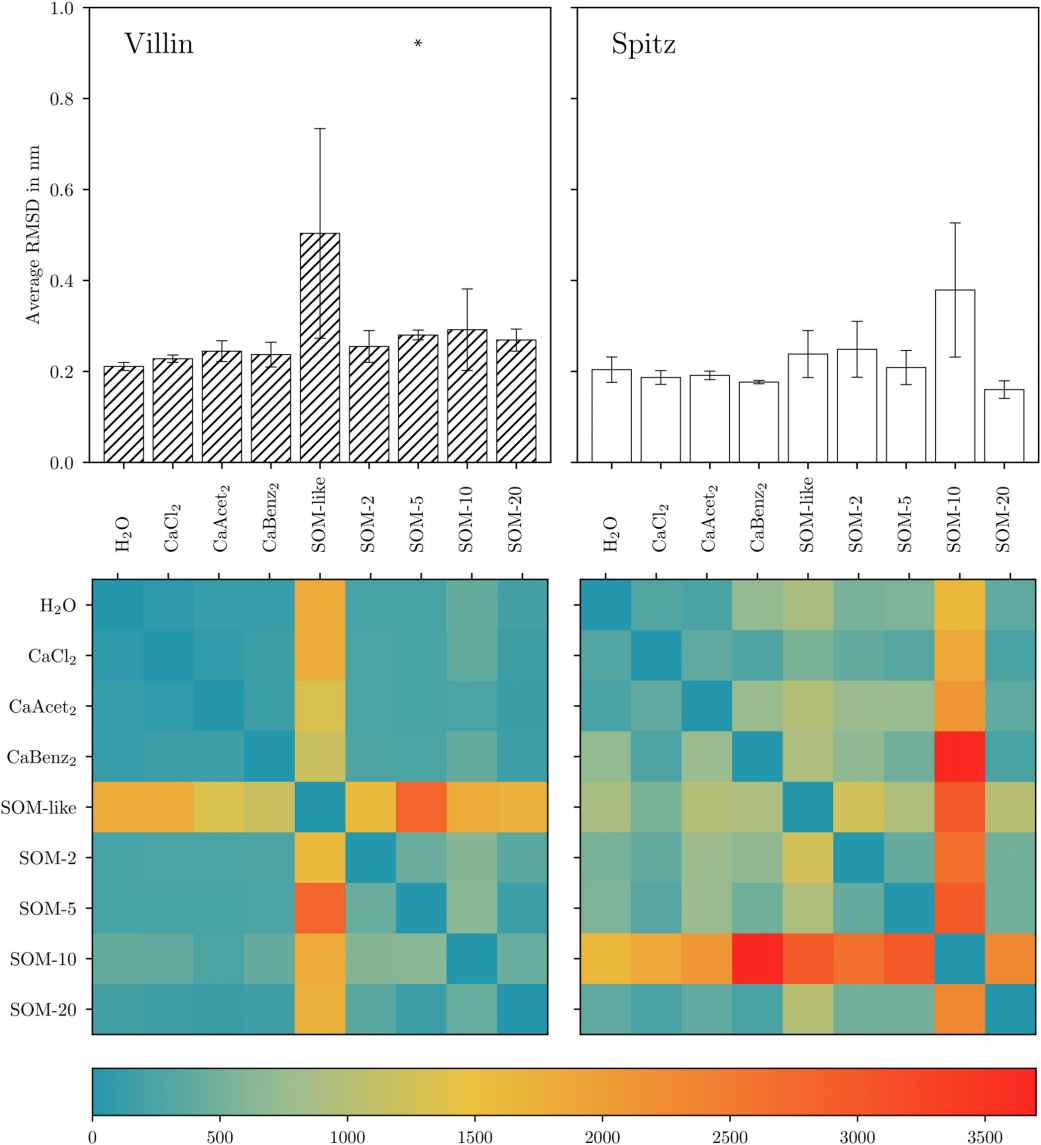
Average RMSD over simulation time and replicates (top panels) and heatmaps of the pairwise *D*_*HES*_ values (bottom panels) for villin (left panels) and spitz (right panels), respectively. The error bars of the RMSD represent the SD between replicates. Significant differences to the respective H_2_O simulations are indicated [Color figure can be viewed at wileyonlinelibrary.com]

**FIGURE 3:**
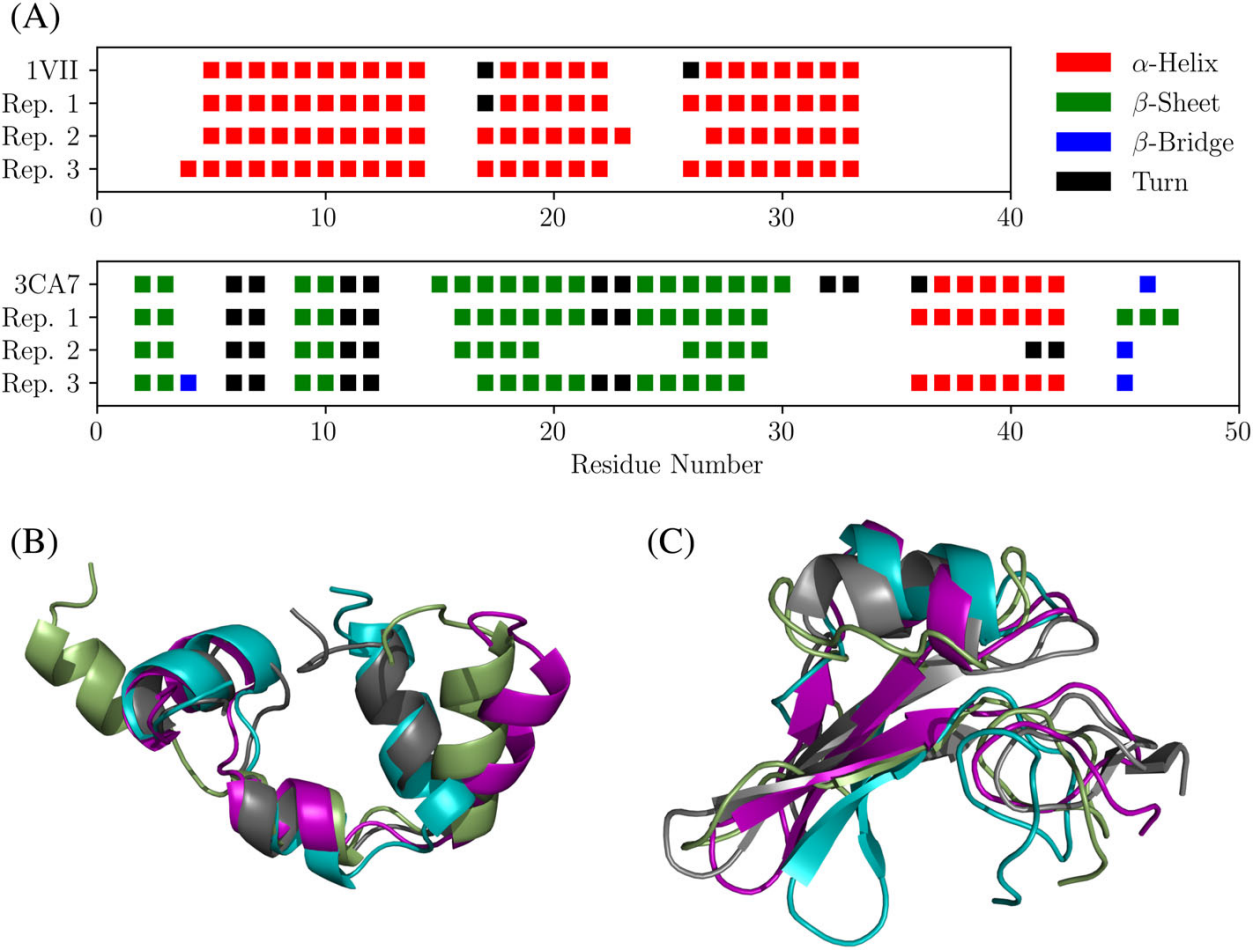
A, Secondary structures of villin (top panel) and spitz (bottom panel) per amino acid residue as assigned by DSSP.^38^ For both proteins the PDB reference and the endpoint of the trajectories of conditions in which unfolding events were detected (SOM‐like and SOM‐10 for villin and spitz, respectively) were plotted. B, Reference molecular structure of villin from PDB (gray) and SOM‐like replicates 1, 2, and 3 (purple, smudge green, and teal, respectively) in the trajectory endpoint. C, Reference molecular structure of spitz from PDB (gray) and SOM‐10 replicates 1, 2, and 3 (purple, smudge green, and teal, respectively) in the trajectory endpoint. The molecular structures were rendered with PyMol.^42^ [Color figure can be viewed at wileyonlinelibrary.com]

The backbone root‐mean‐square deviation (RMSD) and the pairwise harmonic ensemble similarity (*D*_*HES*_) for both reference proteins in all simulated conditions were analyzed to measure the protein stability and conformational changes between different conditions, respectively (see Figure 2 and Tables S1 and S2). For villin the highest deviations of protein stability to H_2_O were seen in SOM‐like. In this condition two of three replicates unfolded, which was reflected by a high RMSD and a high *D*_*HES*_. To show the extent of these unfolding events the endpoints of the simulated trajectories and the PDB structure of villin are shown in Figure 3B. Additionally, in Figure 3A (top panel) the secondary structure assigned to every amino acid residue of the same snapshots is depicted. It is clearly visible that the increase in RMSD is due to the movement of the three *α*‐helices with respect to each other, while these secondary structure elements remain intact. Interestingly, the more complex systems created by VSOMM (SOM‐2, SOM‐5, SOM‐10, SOM‐20) lead to slightly increased RMSD values, but no major unfolding event was observed. The average RMSD of villin in SOM‐5 was significantly increased compared with H_2_O (two‐sample *t*‐test, *P* = .017). Note however that such a small difference in RMSD, together with a low *D*_*HES*_ suggests that the villin headpiece remains strcuturally unaffected in the SOM‐5 system. In contrast to villin, spitz was more stable in the SOM‐like environments. Moreover, the average RMSD of spitz did not significantly deviate from the value observed in H_2_O in any of the tested conditions. The highest average RMSD of spitz was observed in SOM‐10 (0.38 ± 0.15 compared with 0.20 ± 0.03 in H_2_O and 0.26 ± 0.06 from literature^40^), where also the highest conformational variability (*D*_*HES*_) was found. In Figure 3A (bottom panel) and 3C, the secondary structure assignments and the molecular conformation of the endpoints of the simulation trajectories of spitz in SOM‐10 are shown. Interestingly, while the protein kept its overall fold and compactness, partial loss of secondary structure (both *α*‐helix and *β*‐sheet) was observed for replicates 2 and 3.

However, the secondary structures depicted in Figure 3A were just trajectory endpoint snapshots. To gain a broader insight the average fractions of secondary structure elements for both proteins in all conditions were analyzed (Table S3). Even for the simulations with the highest RMSD and D_*HES*_, villin's secondary structure was remarkably stable. Similarly, secondary structure of spitz remained stable in all simulated systems, with the exception of the SOM‐10 condition, where partial loss was detected as pointed out above. For both proteins, no significant reduction of secondary structure in any condition compared with water was observed.

The *D*_*HES*_ analysis (Figure 2, bottom panels) not only shows higher pairwise values between VSOMM2 systems and H_2_O, compared with simple co‐solvent conditions, but also higher values within the SOM systems themselves (higher values in the fourth (+ −) quadrant than in the second (− +) quadrant). This indicates that the presence of more complex SOM molecules leads to a higher conformational variability in both proteins.

Root‐mean‐square fluctuations (RMSF) of C*α* atoms can give additional insight into the rigidity of the proteins. Similarly to the RMSD and D_*HES*_ analysis the RMSF comparison of villin and spitz in different SOM models are remarkably similar to their behavior in H_2_O (Figures S1 and S2). There were two exceptions to this rule, villin in SOM‐like and spitz in SOM‐10, which is in line with the results above. Interestingly, for villin in SOM‐like the mean RMSF is clearly increased compared with the H_2_O mean RMSF indicating an increase in fluctuations. Contrarily, this is not the case for spitz in SOM‐10, where the mean RMSF does not differ from the water reference to such an extent, with a high deviation observed in only one replicate.

### 3.2 Protein‐solvent interactions

#### 3.2.1 Interaction energies

**FIGURE 4:**
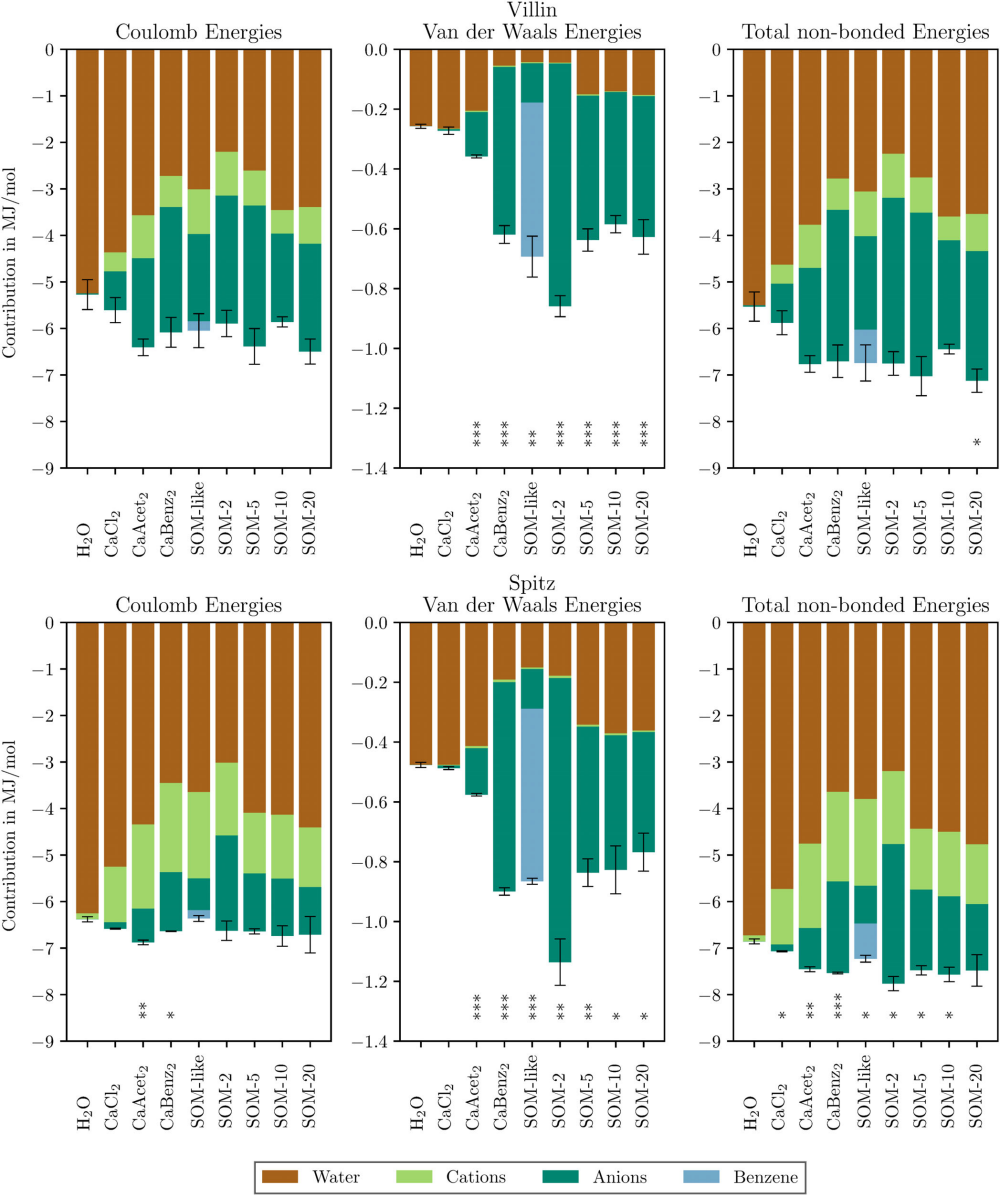
Nonbonded interaction energies of proteins with their surroundings observed in different systems. Significant differences to the respective H_2_O simulations are indicated. The different colors represent the average contribution of a solvent molecule type. Error bars are SEs of the mean over three independent replicates. Note that y‐axes have different scales [Color figure can be viewed at wileyonlinelibrary.com]

The nonbonded interaction energies were investigated to understand which forces govern the interaction of proteins and their surroundings. The interactions were grouped according to their respective (co‐)solvent components to gather more insight. In Figure 4, the nonbonded interaction energies between (co‐)solvent molecules and the proteins are depicted. For all simulated conditions, the Coulombic interaction energies were larger than the respective van der Waals energies by approximately a factor of 10 (compare Figure 4, left and middle panels). Additionally, differences in the contribution of negatively charged SOM molecules and positively charged Ca^2+^ ions can be seen between the two reference proteins (Figure 4 left panels, compare size of light green bars between villin and spitz). Moreover, the contribution of water to the interaction energies to the protein was reduced when organic co‐solvent molecules were present. However, only in a few conditions the addition of SOM molecules led to significantly more favorable Coulombic interactions (Figure 4, left panels). In contrast, the addition of SOM molecules led to considerably stronger van der Waals interaction energies in all simulations (Figure 4, middle panels). Overall, the strength of the total nonbonded interaction energies between protein and their surroundings increased consistently with the addition of SOM molecules (Figure 4, right panels).

#### 3.2.2 Spatial arrangement of co‐solvent molecules

**FIGURE 5:**
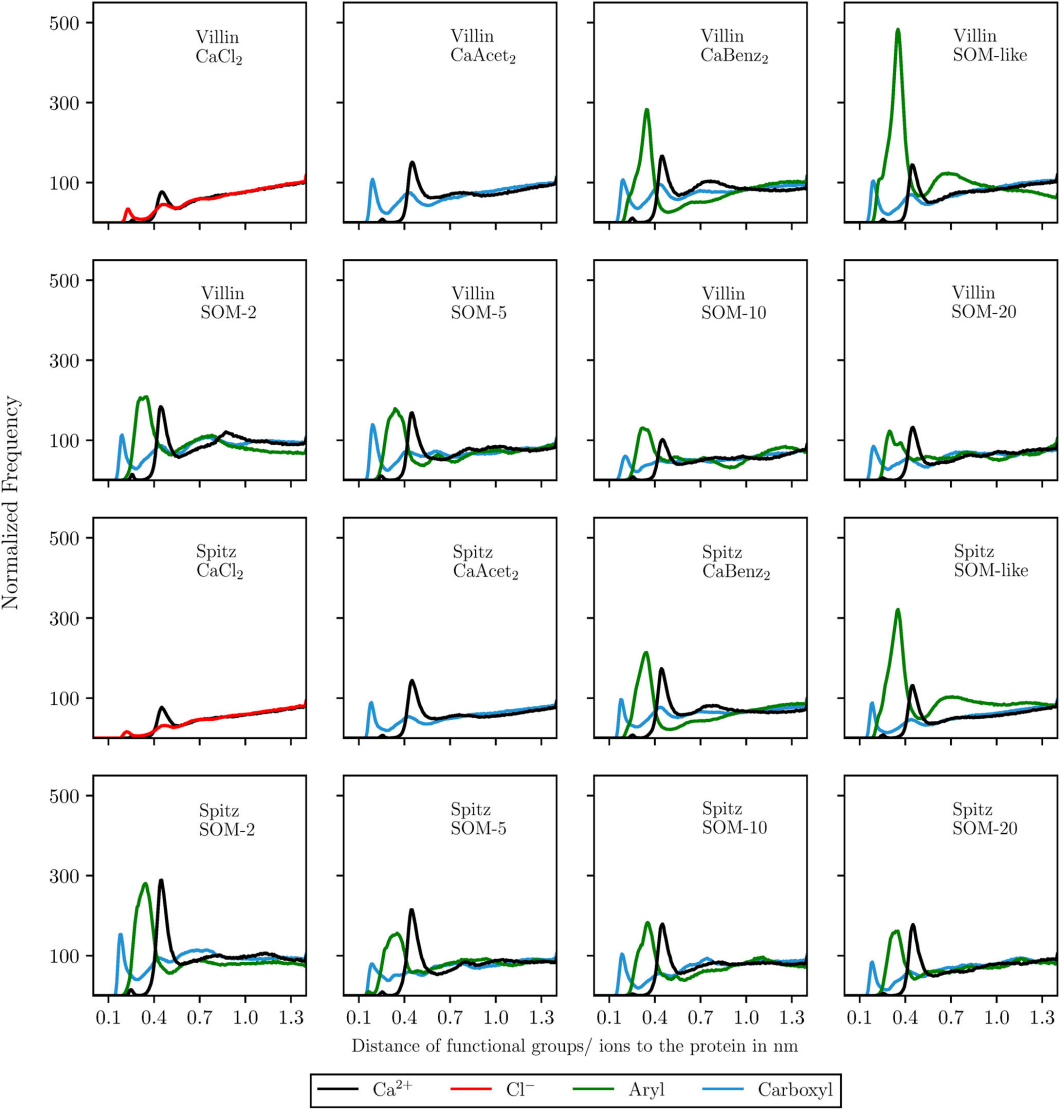
Normalized frequency of the minimum distances of selected functional groups/ions to the reference proteins. The frequency is counted as the number of snapshots sampled at a given distance over all replicates. Normalization was done over the number of functional groups/ions present in the systems [Color figure can be viewed at wileyonlinelibrary.com]

To understand which kind of molecular interface a protein is experiencing in different SOM model systems, the proximity of ions and functional groups to the protein is of interest. Thus, a minimum distance function (MDF) was calculated, transformed to a histogram, normalized, and finally plotted (Figure 5). The highest peak of Ca^2+^, which was present in all shown simulations, ranged around 0.44 to 0.45 nm distance to the protein. Interestingly, for both reference proteins the negatively charged carboxyl groups (blue line) were in close proximity to the protein with maxima ranging from 0.18 to 0.20 nm. A second distinct peak of carboxyl groups was found between 0.43 and 0.45 nm. The aromatic peak (green line) was always found in between the peak for carboxyl groups and Ca^2+^ ions with maxima between 0.29 and 0.35 nm. Strikingly, even in systems where benzoate was present and thus both carboxyl and aryl groups were on the same molecule, the maxima of the peaks did not differ from other simulations.

#### 3.2.3 Hydrogen bonds

**FIGURE 6:**
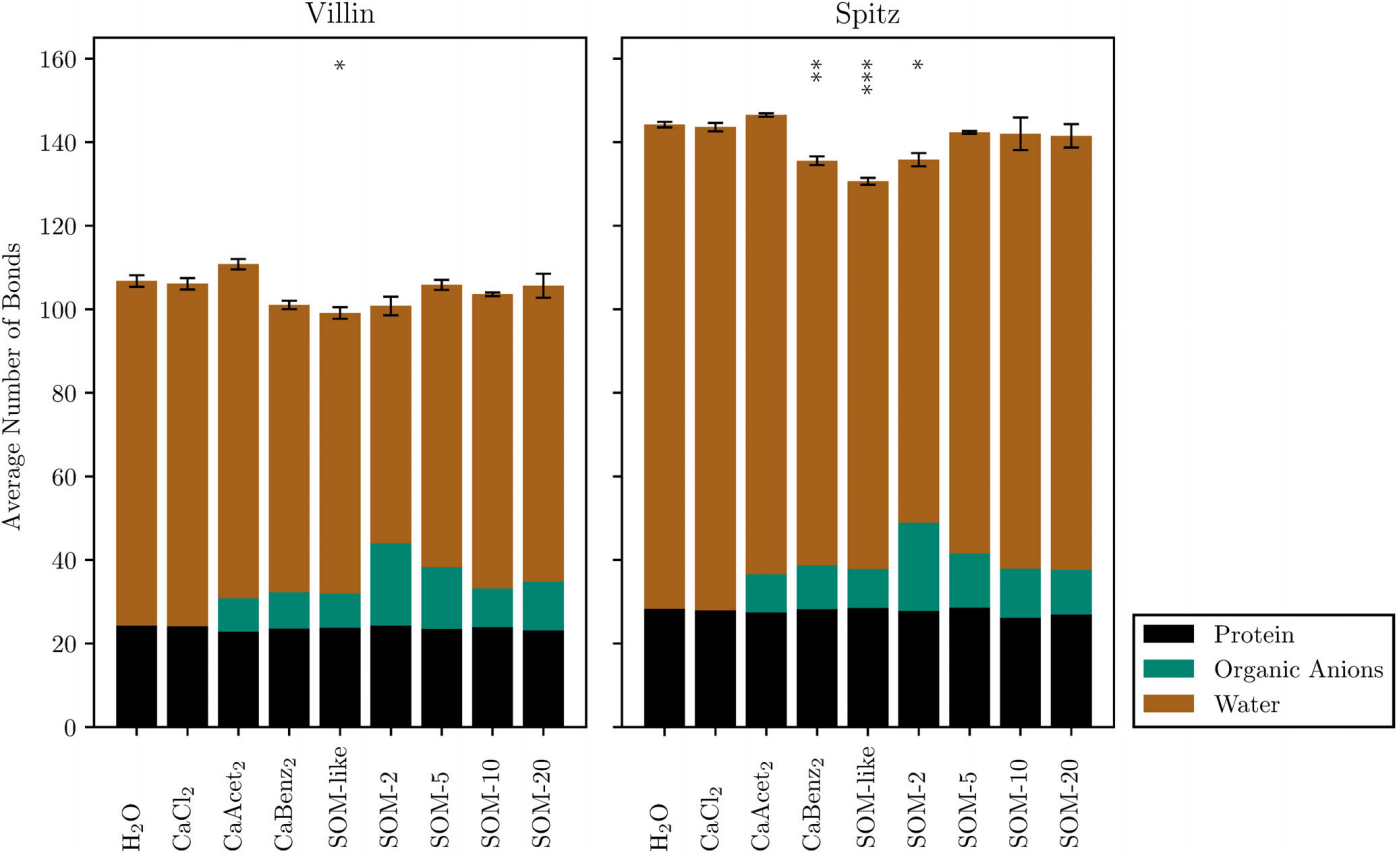
Average number of hydrogen bonds formed by the reference proteins over the simulation. The total bar heights and their respective error bars represent all hydrogen bonds formed by the protein and their SD over the replicates. The different colors indicate the hydrogen bond partners. No differentiation was made between hydrogen bond donors and acceptors [Color figure can be viewed at wileyonlinelibrary.com]

Since the MDF analysis indicated that carboxyl groups of co‐solvent molecules are in close contact with the protein, the hydrogen bonds formed by proteins were monitored and averaged over the simulated trajectories. Figure 6 depicts the number of hydrogen bonds formed by villin and spitz with themselves, organic anions and water molecules. In H_2_O simulations, the average number of hydrogen bonds formed by the proteins were 106.8 ± 1.4 and 144.2 ± 0.7 for villin and spitz, respectively. In SOM‐like systems the number of hydrogen bonds formed by both proteins decreased significantly. For most other conditions, however, no significant changes were observed. In general, there are two main observations to be made. First, the total number of hydrogen bonds formed within the protein (black bars) did not change in any condition. Second, hydrogen bonds formed by water molecules are replaced by hydrogen bonds between SOM molecules and the proteins (dark green bars).

### 3.3 Phase separation

#### 3.3.1 Preferential solvation

In order to approach the phase separation observed in some systems, we calculated the preferential solvation between different species in each of the simple co‐solvent conditions via the Kirkwood‐Buff integrals. Values of preferential solvation (*δ*) close to zero indicate a homogeneous mixing of the species in CaCl_2_, CaAcet_2_, and CaBenz_2_ (Table S4‐S6). The calculated *δ* values for SOM‐like, however, indicate phase separation constituted by an organic phase (benzene molecules) and an aqueous phase (water molecules and ionic species) not present in any of the other simple co‐solvent conditions (Table S7). For an analysis and discussion of the preferential solvation of models created with VSOMM2, we refer to Escalona et al.^23^

#### 3.3.2 Formation of clusters

To further the analysis of the phase separation described above and to quantify the extend of aggregation behavior of SOM molecules, we performed cluster analyses on all tested conditions that contained organic co‐solvent molecules. Two molecules were considered to be part of the same cluster if they were connected by at least one hydrogen bond.

**FIGURE 7:**
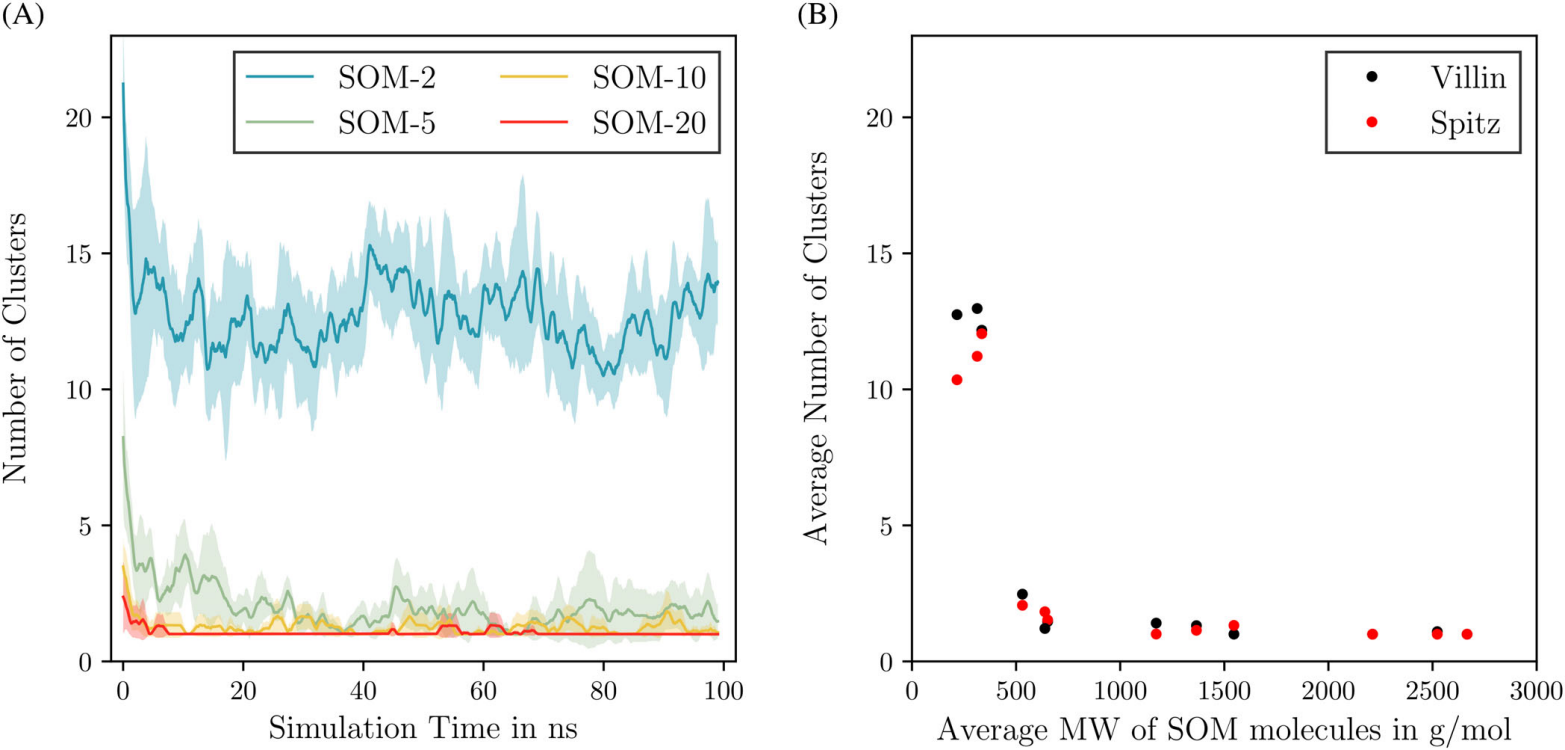
A, Running average (1 ns) time series of the number of clusters formed by humic substance molecules and villin. The colors represent the different sizes of the LHA molecules and the filled area represents the SE of the respective three replicates. B, The average number of clusters of the last 80 ns of the simulation is plotted against the average molecular weight per SOM molecule for both reference proteins [Color figure can be viewed at wileyonlinelibrary.com]

Figure 7A depicts the number of clusters made of LHA molecules and villin as a function of simulation time. After an initial random distribution of molecules, the number of clusters quickly decreased until a lower limit is reached, where it stayed roughly constant. Figure 7B shows the average number of clusters of the last 80 ns of simulation. Strikingly, the net charge of the reference proteins did not considerably influence the clustering of SOM molecules around the protein. No formation of clusters was observed for conditions with simple SOM models (Figure S3).

## 4 DISCUSSION

Two small reference proteins (villin and spitz) were examined in different model SOM environments (starting from simple co‐solvent molecules and progressing to more complex soil organic matter models by VSOMM2) by means of molecular dynamics simulation. We analyzed them in terms of their structural stability, the nature and strength of the interactions with their surroundings as well as their aggregation behavior.

Even though the proteins were exposed to relatively harsh environments in terms of high salt concentration, presence of organic acids, and aromatic compounds, structural stability was mostly maintained. Although the average RMSD of villin in SOM‐5 showed significant difference to villin in water, no unfolding nor changes in other structure‐related properties (conformational variability *D*_*HES*_, secondary structure or RMSF) were observed for this system. In addition, such a small increase in RMSD from 0.21 to 0.28 nm (a value comparable to the average RMSD of 0.27 ± 0.12 nm of villin in water reported in Reference 25), indicates that the protein remains structurally unaffected in the SOM‐5 environment. The only unfolding events were observed for villin in the SOM‐like condition, through the disruption of the hydrophobic core while the second structure remained intact. Interestingly, this coincided with a phase separation between an aqueous and an organic phase, potentially playing an important role in unfolding, since the stability of villin primarily results from hydrophobic interactions within its core. Similarly, the stability of spitz in most of the SOM systems was not affected, with the only exception being the SOM‐10 condition. Here, partial unfolding was detected primarily in terms of the secondary structure loss, with the overall fold and the compactness of the remaining intact, most probably due to the additional disulfide bridges it contains. Interestingly, in both cases, despite the substantial increase in the average RMSD (0.50 and 0.38 nm, respectively) comparing to the water systems (0.21 and 0.20 nm, respectively), no statistical significance was found, probably due to a small sample size (n = 3). Note however, that the observed differences in combination with other structure‐related analyses performed in this study (Figures 2, 3, S1, S2 and Tables S2 and S3), clearly show unfolding of villin and spitz in the SOM‐like and the SOM‐10 environments, respectively, even though these RMSD differences remain statistically insignificant.

In general, we observed differences in protein structure and stability in SOM models compared with water. However, it is important to point out that these changes were not detrimental to the secondary structure and that the dynamics of both proteins stayed unperturbed in almost all simulated conditions. This would indicate that proteins are not only chemically protected as described by Zang et al,^41^ but that they can also retain structure‐related functions.

The analysis of interactions between protein and surrounding SOM molecules showed that they are mainly governed by electrostatic (Coulombic) forces. This is in accordance with recent experimental studies which found that electrostatic forces drive the encapsulation of positively charged proteins with humic substances at pH 5 to 8.^6^ Nonetheless, the weaker van der Waals forces also had a nonnegligible impact. For example, their increased strength upon addition of SOM molecules was often responsible for significantly more favorable total nonbonded interactions between protein and its surroundings, when compared with pure water. Interestingly, there were big differences in the van der Waals energies between different sizes of SOM molecules, but not for the Coloumbic forces (compare SOM‐2 and SOM‐10 conditions in Figure 4). This can be explained by two factors. First, for small molecules it is easier to align in a way so that electrostatic and van der Waals energies are optimized. Second, the electrostatic interactions are longer ranged and, therefore, do not decrease as rapidly as van der Waals interaction when the molecular alignment is not perfect.

To understand how close co‐solvent functional groups and ions get to the protein, minimum distance functions were calculated. The results answered the question of which kind of molecular interface a protein was experiencing in different solvents. Interestingly, even though the composition of co‐solvent molecules changed drastically, the characteristic peaks of the functional groups stayed at constant distances from the reference proteins. This indicated that, even though there is high variability in the arrangement of functional groups within the co‐solvent molecules, the proteins were experiencing a relatively similar solvent interface altogether. This low variations of the interface might be the reason for relatively high stability of proteins in the harsh SOM systems. Moreover, the proximity of carboxyl groups to the protein emphasizes their importance for the interaction of protein and solvent, often as part of hydrogen bonds. Consequently, a high number of hydrogen bonds formed by carboxyl groups and the protein was observed. The fact that the average number of hydrogen bonds formed within the protein is not disturbed by the addition of SOM molecules is in agreement with the observation that the secondary structure of the proteins was not significantly negatively influenced. Preferential solvation calculations showed that the phase separation coincides with a higher variation of RMSD values for villin. This indicates that phase separation, which is mainly driven by hydrophobic forces, has the potential to disturb protein stability.

Cluster analysis showed that, although Leonardite humic acid molecules were initially placed at random positions after approximately 20 ns of simulation time, they associated with the protein and each other to form few yet large clusters. The average number of clusters that formed in a given system depended heavily on the size of SOM molecules, which is explained by the fact that if there are fewer and larger molecules present, fewer hydrogen bonds need to be formed to create a large cluster. The quick formation of protein‐LHA aggregates indicated that proteins are likely to be absorbed by soil organic matter. Interestingly, in a range of +2 (villin) to −2 (spitz), the net charge of a protein had no observable influence on the formation of protein‐SOM clusters.

## 5 CONCLUSION

To the best of our knowledge, this study is the first that tries to explain interactions of protein and SOM at a molecular level by means of molecular dynamics simulations. Most importantly, an association of SOM molecules and proteins to clusters was observed, which seems to have little to no effect on protein stability and secondary structure. These findings lead to the conclusion that SOM can act as a natural reservoir of proteins, which may result in lowered biological accessibility, for example by degrading enzymes. However, protein functionality could remain intact. Our observations are also relevant for the use of enzymes in bioremediation projects, since care needs to be taken so that such enzymes will sustain their activity and accessibility to the intended substrates. While the SOM environment seems to have limited effect on the protein structure, for these two simple proteins an encapsulation by SOM may hamper an efficient use in bioremediation. However, it should be kept in mind that in this study the protein molecules were treated as homogeneous objects, despite different amino acids conferring very different chemical properties. Therefore, we suggest further investigation of individual amino acids and their interactions with SOM in future studies.

### PEER REVIEW

The peer review history for this article is available at https://publons.com/publon/10.1002/prot.26070.

## Supporting information

**APPENDIX S1**: Supplementary Information

## Data Availability

The data that support the findings of this study are available from the corresponding author upon reasonable request.
