# Supplementary material for: Exploring the structure and dynamics of proteins in soil organic matter
Source: Proteins. 2021 Mar 25;89(8):925–36. doi: 10.1002/prot.26070 (PMC8360018; doi:10.1002/prot.26070)
Supplement: Supplementary file 1 — APPENDIX S1: Supplementary Information [file PROT-89-925-s001.pdf]

Supplementary Information for

Exploring the structure and dynamics of proteins in soil organic matter

Mathias Gotsmy, Yerko Escalona, Chris Oostenbrink & Drazen Petrov

Department of Material Sciences and Process Engineering, Institute of Molecular Modeling and Simulation, University of Natural Resources and Life Sciences Vienna, Vienna, Austria

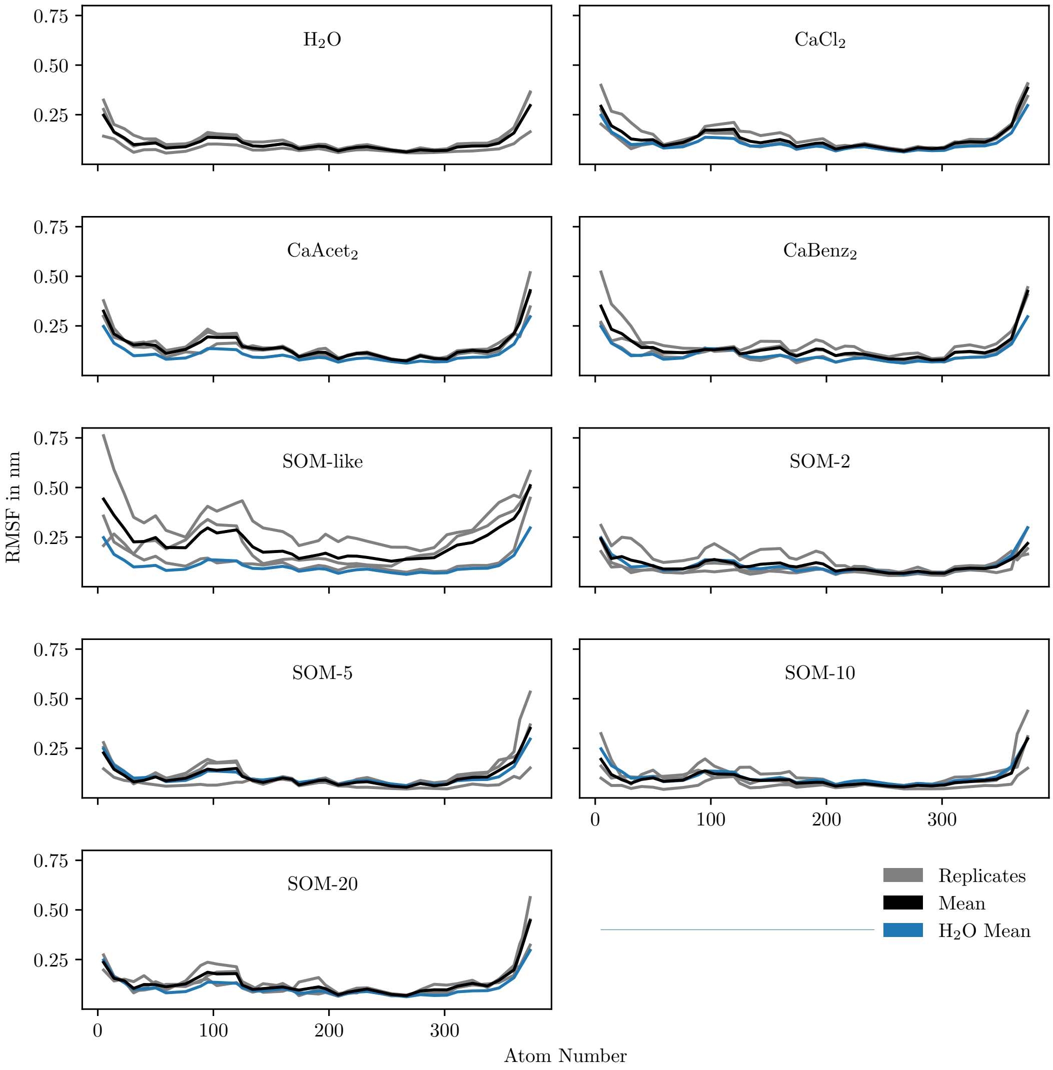

Figure S1: Root mean square fluctuation (RMSF) analysis of villin in simulated conditions. As a reference the mean RMSF of the H_2O condition was plotted in the other conditions.

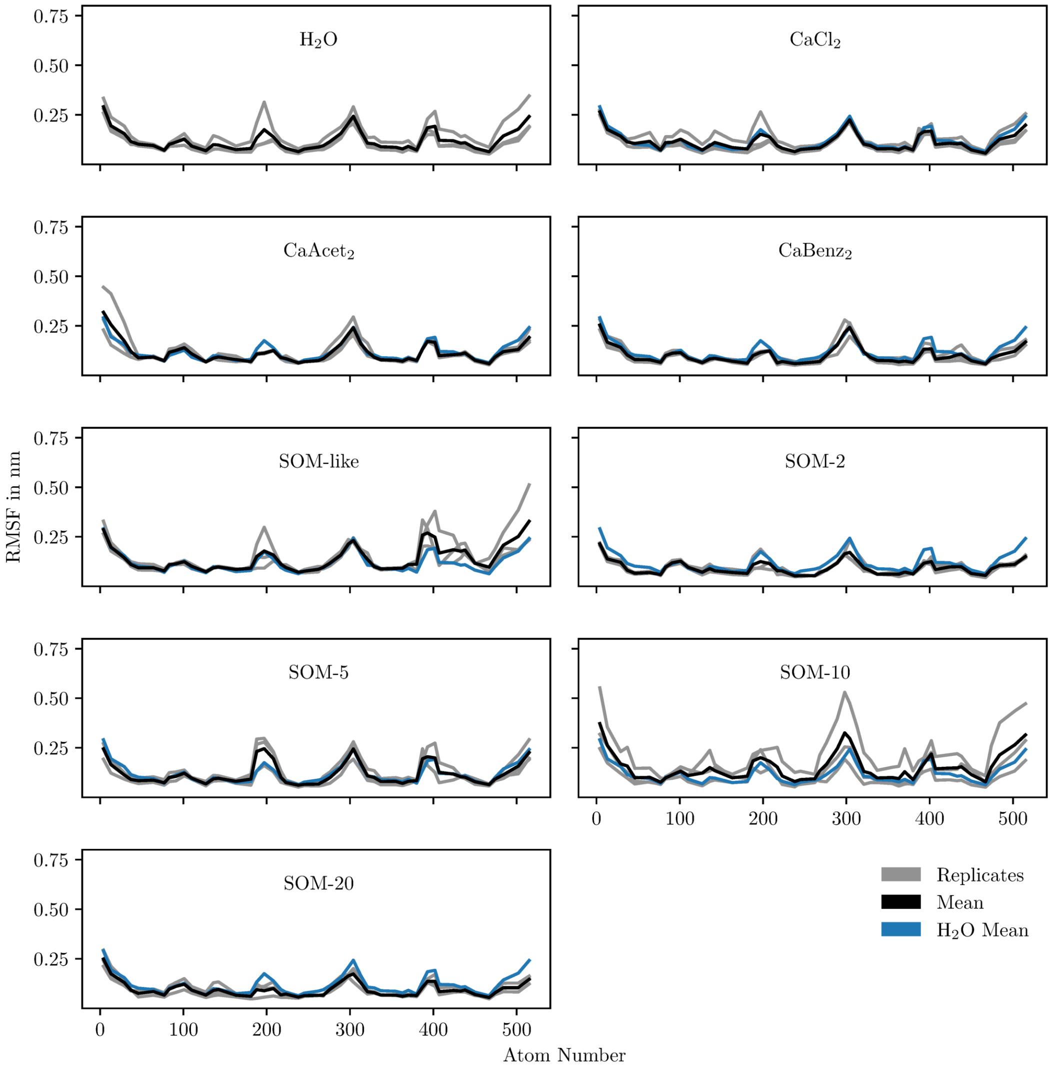

Figure S2: Root mean square fluctuation (RMSF) analysis of spitz in simulated conditions. As a reference the mean RMSF of the H_2O condition was plotted in the other conditions.

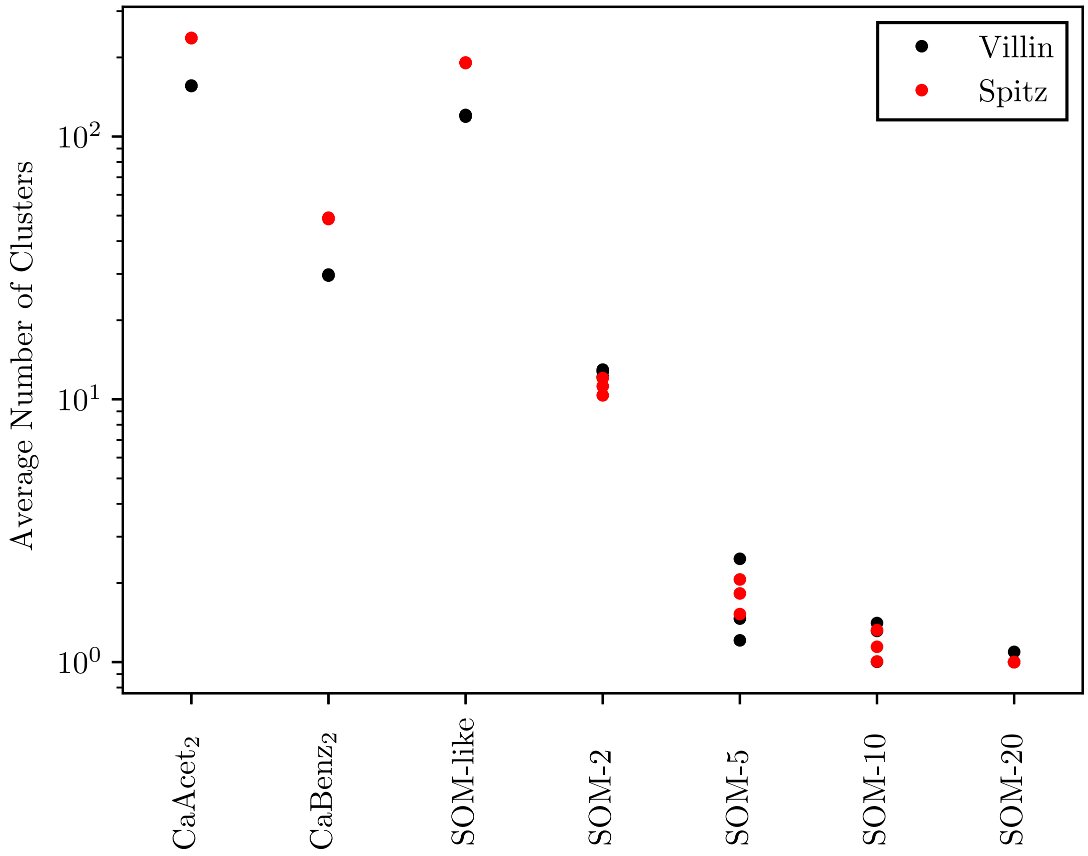

Figure S3: The average number of clusters of the last 80 ns of the simulation plotted for both proteins. In $CaAcet_2$, $CaBenz_2$ and SOM-like only very small and short-lived clusters are formed and, therefore, the number of clusters is primarily depending on the number of co-solvent molecules in the system. Since simple solvent systems for spitz were bigger than for villin and the co-solvent molecule concentration was equal, the average number of clusters for spitz was higher. Another consequence of this is that for said conditions it appears that there was only one point per protein — the average number of clusters for all replicates is almost identical.

Table S1: Average RMSDs of the simulation in nm. SOM-5 conditions are significantly different to H_2O for villin (two-sample t-test, $p = 0.017$).

	Rep.	H_2O	$CaCl_2$	$CaAcet_2$	$CaBenz_2$	SOM-like	SOM-2	SOM-5	SOM-10	SOM-20
Villin	1	0.22	0.22	0.23	0.27	0.48	0.28	0.29	0.22	0.30
	2	0.20	0.23	0.23	0.21	0.80	0.21	0.27	0.24	0.25
	3	0.21	0.24	0.28	0.23	0.23	0.27	0.28	0.42	0.25
Mean		0.21	0.23	0.24	0.24	0.50	0.25	0.28	0.29	0.27
Std. Dev.		0.01	0.01	0.02	0.03	0.23	0.03	0.01	0.09	0.02
Spitz	1	0.24	0.18	0.19	0.17	0.31	0.22	0.18	0.18	0.17
	2	0.18	0.21	0.18	0.17	0.20	0.33	0.26	0.41	0.13
	3	0.19	0.17	0.20	0.18	0.20	0.19	0.19	0.54	0.17
Mean		0.20	0.19	0.19	0.18	0.24	0.25	0.21	0.38	0.16
Std. Dev.		0.03	0.02	0.01	0.00	0.05	0.06	0.04	0.15	0.02

Table S2: Pairwise D_{HES} values in simulated conditions. Errors were estimated by bootstrapping.

Villin	H_2O	$CaCl_2$	$CaAcet_2$	$CaBenz_2$	SOM-like	SOM-2	SOM-5	SOM-10	SOM-20
H_2O	0 ± 0	65 ± 3	124 ± 3	128 ± 2	1783 ± 46	221 ± 2	225 ± 4	413 ± 8	204 ± 2
$CaCl_2$	65 ± 3	0 ± 0	87 ± 1	153 ± 2	1788 ± 38	252 ± 2	221 ± 4	394 ± 6	173 ± 2
$CaAcet_2$	124 ± 3	87 ± 1	0 ± 0	147 ± 2	1329 ± 25	251 ± 3	220 ± 4	247 ± 3	139 ± 2
$CaBenz_2$	128 ± 2	153 ± 2	147 ± 2	0 ± 0	1173 ± 18	272 ± 2	257 ± 3	410 ± 6	170 ± 2
SOM-like	1783 ± 46	1788 ± 38	1329 ± 25	1173 ± 18	0 ± 0	1601 ± 28	2798 ± 58	1807 ± 33	1715 ± 33
SOM-2	221 ± 2	252 ± 2	251 ± 3	272 ± 2	1601 ± 28	0 ± 0	435 ± 5	625 ± 8	345 ± 3
SOM-5	225 ± 4	221 ± 4	220 ± 4	257 ± 3	2798 ± 58	435 ± 5	0 ± 0	650 ± 12	151 ± 2
SOM-10	413 ± 8	394 ± 6	247 ± 3	410 ± 6	1807 ± 33	625 ± 8	650 ± 12	0 ± 0	431 ± 6
SOM-20	204 ± 2	173 ± 2	139 ± 2	170 ± 2	1715 ± 33	345 ± 3	151 ± 2	431 ± 6	0 ± 0
**Spitz**	H_2O	$CaCl_2$	$CaAcet_2$	$CaBenz_2$	SOM-like	SOM-2	SOM-5	SOM-10	SOM-20
H_2O	0 ± 0	290 ± 6	232 ± 5	710 ± 13	891 ± 18	543 ± 6	578 ± 13	1606 ± 26	382 ± 8
$CaCl_2$	290 ± 6	0 ± 0	383 ± 6	271 ± 5	558 ± 11	403 ± 5	317 ± 4	1860 ± 30	240 ± 4
$CaAcet_2$	232 ± 5	383 ± 6	0 ± 0	753 ± 13	980 ± 16	767 ± 11	742 ± 15	2106 ± 30	377 ± 6
$CaBenz_2$	710 ± 13	271 ± 5	753 ± 13	0 ± 0	901 ± 14	684 ± 9	490 ± 7	3666 ± 56	245 ± 3
SOM-like	891 ± 18	558 ± 11	980 ± 16	901 ± 14	0 ± 0	1251 ± 17	953 ± 17	2963 ± 52	1002 ± 15
SOM-2	543 ± 6	403 ± 5	767 ± 11	684 ± 9	1251 ± 17	0 ± 0	406 ± 5	2631 ± 36	497 ± 5
SOM-5	578 ± 13	317 ± 4	742 ± 15	490 ± 7	953 ± 17	406 ± 5	0 ± 0	2969 ± 43	489 ± 7
SOM-10	1606 ± 26	1860 ± 30	2106 ± 30	3666 ± 56	2963 ± 52	2631 ± 36	2969 ± 43	0 ± 0	2301 ± 34
SOM-20	382 ± 8	240 ± 4	377 ± 6	245 ± 3	1002 ± 15	497 ± 5	489 ± 7	2301 ± 34	0 ± 0

Table S3: Secondary Structure fractions of reference proteins according to DSSP. No significant loss of secondary structure compared to H_2O was observed.

Villin	Rep.	H_2O	$CaCl_2$	$CaAcet_2$	$CaBenz_2$	SOM-like	SOM-2	SOM-5	SOM-10	SOM-20
A-Helix	1	0.643	0.646	0.637	0.670	0.637	0.67	0.636	0.643	0.598
	2	0.641	0.644	0.635	0.665	0.660	0.646	0.655	0.649	0.608
	3	0.651	0.657	0.654	0.648	0.665	0.594	0.657	0.636	0.643
B-Bridge	1	0.000	0.000	0.000	0.000	0.000	0.000	0.000	0.000	0.001
	2	0.000	0.000	0.000	0.000	0.000	0.000	0.000	0.000	0.000
	3	0.000	0.000	0.000	0.000	0.000	0.000	0.000	0.000	0.000
B-Sheet	1	0.000	0.000	0.000	0.000	0.000	0.000	0.000	0.000	0.000
	2	0.000	0.000	0.000	0.000	0.000	0.000	0.000	0.000	0.000
	3	0.000	0.000	0.000	0.000	0.000	0.000	0.000	0.000	0.000
Turn	1	0.051	0.044	0.049	0.030	0.042	0.019	0.038	0.033	0.085
	2	0.044	0.033	0.056	0.026	0.058	0.039	0.035	0.041	0.083
	3	0.040	0.037	0.044	0.035	0.027	0.067	0.032	0.038	0.050
Structure	1	0.694	0.690	0.687	0.701	0.679	0.689	0.674	0.676	0.683
	2	0.685	0.677	0.690	0.692	0.718	0.685	0.690	0.690	0.690
	3	0.691	0.694	0.699	0.683	0.692	0.660	0.690	0.674	0.693
Spitz	Rep.	H_2O	$CaCl_2$	$CaAcet_2$	$CaBenz_2$	SOM-like	SOM-2	SOM-5	SOM-10	SOM-20
A-Helix	1	0.139	0.137	0.003	0.140	0.140	0.139	0.137	0.139	0.139
	2	0.139	0.128	0.130	0.140	0.140	0.110	0.145	0.000	0.084
	3	0.002	0.139	0.138	0.140	0.139	0.139	0.140	0.124	0.137
B-Bridge	1	0.01	0.005	0.015	0.003	0.01	0.002	0.003	0.004	0.003
	2	0.006	0.009	0.005	0.003	0.005	0.004	0.010	0.030	0.006
	3	0.007	0.004	0.013	0.005	0.013	0.006	0.004	0.020	0.007
B-Sheet	1	0.320	0.358	0.331	0.365	0.263	0.326	0.378	0.374	0.378
	2	0.352	0.348	0.363	0.364	0.390	0.339	0.325	0.258	0.365
	3	0.351	0.333	0.316	0.376	0.343	0.407	0.350	0.250	0.302
Turn	1	0.103	0.104	0.242	0.082	0.090	0.114	0.083	0.102	0.098
	2	0.095	0.136	0.125	0.127	0.110	0.168	0.107	0.153	0.179
	3	0.235	0.089	0.106	0.095	0.105	0.114	0.093	0.109	0.097
Structure	1	0.572	0.605	0.591	0.591	0.503	0.580	0.600	0.620	0.618
	2	0.593	0.621	0.624	0.634	0.645	0.621	0.588	0.441	0.633
	3	0.595	0.565	0.574	0.616	0.601	0.666	0.587	0.503	0.543

Table S4: Preferential solvation δ values of the $CaCl_2$ solvent only simulation. The left column refers to the center from where the radial distribution functions were calculated.

	H_2O	Ca^{2+}	Cl^-
H_2O	0.000	0.000	0.000
Ca^{2+}	-0.001	-0.002	0.003
Cl^-	-0.002	0.002	0.000

Table S5: Preferential solvation δ values of the $CaAcet_2$ solvent only simulation. The left column refers to the center from where the radial distribution functions were calculated.

	H_2O	Ca^{2+}	$Acetate^-$
H_2O	0.000	0.000	0.000
Ca^{2+}	0.001	-0.002	0.001
$Acetate^-$	0.001	0.001	-0.002

Table S6: Preferential solvation δ values of the $CaBenz_2$ solvent only simulation. The left column refers to the center from where the radial distribution functions were calculated.

	H_2O	Ca^{2+}	$Benzoate^-$
H_2O	0.000	0.000	0.000
Ca^{2+}	0.000	-0.003	0.003
$Benzoate^-$	0.001	0.001	-0.002

Table S7: Preferential solvation δ values of the SOM-like solvent only simulation. The left column refers to the center from where the radial distribution functions were calculated.

	H_2O	Ca^{2+}	$Acetate^{-}$	$Benzoate^{-}$	Benzene
H_2O	0.003	0.000	0.000	0.000	-0.003
Ca^{2+}	0.005	-0.002	0.002	0.000	-0.004
$Acetate^{-}$	0.004	0.001	-0.002	0.000	-0.003
$Benzoate^{-}$	-0.008	0.001	-0.001	-0.001	0.009
Benzene	-0.025	-0.002	-0.001	0.001	0.026
